# Transcription Factor Binding Site Enrichment Analysis in Co-Expression Modules in Celiac Disease

**DOI:** 10.3390/genes9050245

**Published:** 2018-05-10

**Authors:** Irati Romero-Garmendia, Koldo Garcia-Etxebarria, Hector Hernandez-Vargas, Izortze Santin, Amaia Jauregi-Miguel, Leticia Plaza-Izurieta, Marie-Pierre Cros, Maria Legarda, Iñaki Irastorza, Zdenko Herceg, Nora Fernandez-Jimenez, Jose Ramon Bilbao

**Affiliations:** 1 University of the Basque Country (UPV-EHU) and Biocruces Health Research Institute, 48940 Leioa, Spain; irati.romero@ehu.eus (I.R.-G.); koldo.garcia@biodonostia.org (K.G.-E.); izortze.santingomez@osakidetza.eus (I.S.); amaia.jauregimiguel@gmail.com (A.J.-M.); immunogenetics.let@gmail.com (L.P.-I.); 2 Epigenetics Group, International Agency for Research on Cancer (IARC), 69372 Lyon CEDEX 08, France; hector.hernandez-vargas@lyon.unicancer.fr (H.H.-V.); crosm@iarc.fr (M.-P.C.); hercegz@iarc.fr (Z.H.); 3 Spanish Biomedical Research Center in Diabetes and Associated Metabolic Disorders (CIBERDEM), 28029 Madrid, Spain; 4 Pediatric Gastroenterology Unit, Cruces University Hospital, University of the Basque Country-(UPV/EHU) and Biocruces Health Research Institute, 48903 Barakaldo, Spain; maria.legardatamara@osakidetza.net (M.L.); inakixarles.irastorzaterradillos@osakidetza.net (I.I.)

**Keywords:** celiac disease, complex disease, co-expression, gene regulation, transcription factor

## Abstract

The aim of this study was to construct celiac co-expression patterns at a whole genome level and to identify transcription factors (TFs) that could drive the gliadin-related changes in coordination of gene expression observed in celiac disease (CD). Differential co-expression modules were identified in the acute and chronic responses to gliadin using expression data from a previous microarray study in duodenal biopsies. Transcription factor binding site (TFBS) and Gene Ontology (GO) annotation enrichment analyses were performed in differentially co-expressed genes (DCGs) and selection of candidate regulators was performed. Expression of candidates was measured in clinical samples and the activation of the TFs was further characterized in C2BBe1 cells upon gliadin challenge. Enrichment analyses of the DCGs identified 10 TFs and five were selected for further investigation. Expression changes related to active CD were detected in four TFs, as well as in several of their in silico predicted targets. The activation of TFs was further characterized in C2BBe1 cells upon gliadin challenge, and an increase in nuclear translocation of CAMP Responsive Element Binding Protein 1 (CREB1) and IFN regulatory factor-1 (IRF1) in response to gliadin was observed. Using transcriptome-wide co-expression analyses we are able to propose novel genes involved in CD pathogenesis that respond upon gliadin stimulation, also in non-celiac models.

## 1. Introduction

Celiac disease (CD, MIM: 212750) is a chronic, immune-mediated gastrointestinal disorder that develops in genetically susceptible individuals, due to an inappropriate immune response to ingested gluten. CD affects 1% of Caucasians and results in small intestinal injury and disruption of epithelial dynamics. Intraepithelial lymphocytosis, crypt hyperplasia, villous atrophy and presence of autoantibodies are found in active CD patients [1]. Usually these features revert with a lifelong gluten-free diet (GFD), the only effective treatment for the disease known so far.

CD is a complex disorder in which a combination of genetic and environmental risk factors (mainly ingestion of gluten) drives pathogenesis. The major genetic determinant of CD is associated with HLA class II molecules DQ2 and DQ8, which are necessary but not sufficient for disease development [2]. In the last years, two genome wide association studies (GWAS) and the Immunochip follow-up project have identified additional genetic markers and altogether explain around 50% of the genetic component [3,4,5,6]. 

The majority of the efforts made to understand the functional effects underlying the association signals have been limited to the detection of possible alterations in the expression levels of nearby genes or cis-expression quantitative trait loci (cis-eQTLs), and have yielded limited results [7], indicating a more complex scenario in CD pathogenesis where the coordinated response of groups or pathways of genes is an additional functional layer that is altered. We have previously shown that the expression of *PTPRK* and *THEMIS*, two immune-related genes located on the GWAS peak on hg18: chr6: 127.99–128.38 Mb, was positively correlated in CD patients but not in controls [8]. Moreover, a group of 93 genes related to the NFκB pathway showed a strong correlation of messenger RNA (mRNA) levels in the intestinal mucosa of non-celiac controls that was completely disrupted in active CD [9]. These findings suggest that common genomic regulatory elements such as transcription factors (TFs) could control the expression of groups of genes rather than isolated genes.

TFs are key elements controlling gene expression [10]. Many studies have identified them as drivers of many disease processes such as breast cancer where AGTR2, ZNF132, TFDP3 and others have been identified as regulators [11], or human periodontitis where 41 master regulators have been found [12]. A number of TFs have been implicated in CD; including the master regulator of T cell differentiation BACH2, which is downregulated in CD, while 98 of its targets have altered expression [13]. However, other non-identified TFs could also be contributing to CD pathogenesis, and a transcriptome-wide level search for additional TFs that could also be contributing to CD pathogenesis is justified.

We hypothesize that the presence (or absence) of gluten in individuals that are susceptible to CD could provoke the disruption of groups, or modules, of co-expressed genes, through changes in the function of transcription shared by those modules.

To test this hypothesis, we reanalyzed two expression microarray experiments in which whole-genome expression profiling had been performed to describe the transcriptional program of acute and chronic responses to gluten in the small intestinal mucosa of CD patients [14,15]. In the present work, we constructed co-expression modules at a whole genome level and identified those genes that abandon the modules in response to gliadin, to search for shared TFs that could underlie those changes. We then analyzed the expression of those TFs and several target genes in biopsies from CD patients and controls, as well as nuclear translocation in an in vitro challenged cell culture model. 

## 2. Materials and Methods

### 2.1. Microarray Results and Bioinformatics Analyses

Raw data from two previous Human U133 Plus 2.0 Array (Affymetrix, Santa Clara, CA, USA) experiments were downloaded from the EBI ArrayExpress database (http://www.ebi.ac.uk/microarray-as/ae/; experiment numbers E-MEXP-1828 and E-MEXP-1823). Briefly, the long-term or chronic experiment consisted of duodenal biopsies from nine newly diagnosed, active CD patients and nine treated CD patients on GFD for more than 2 years. In the tissue-culture response experiment 10 biopsies from GFD-CD patients that had been divided into two portions and incubated with (or without) 10 μg/mL gliadin for 4 h were used [14].

The workflow of the study is summarized in Figure S1. Probes from the expression arrays were translated to gene names using the manifest of the array or using BLAST [16] of the probe sequences against the human reference genome Hg19. If a gene was represented by more than one probe, the median of all probe intensities was taken as the expression level of the gene. To prioritize the most informative genes, only those genes whose expression was more variable than the median gene were selected for co-expression analyses, using the “varianceBasedfilter” function in the DCGL R package [17]. Co-expression modules were defined using the weighted correlation network analysis available in the R package WGCNA [18]. In addition, gliadin-induced changes in co-expression were also analyzed, considering differentially co-expressed genes (DCGs) as those genes that change significantly their relationships with the rest of the genes in their module, when comparing gliadin-stimulation (active CD or in vitro gliadin-challenge) to its respective basal situation, and viceversa. DCGs were identified using the DCGL R package and those with nominal *p*-value < 0.05 were considered.

### 2.2. Transcription Factor Binding Site and Gene Ontology Enrichment Analysis

DCGs within each co-expression module were compared to the rest of the genome in order to detect significantly overrepresented transcription factor binding sites (TFBS), using the FatiGO tool [19] available in the Babelomics v4.3 (http://v4.babelomics.org/) suite from the CIPF Research Center in Valencia, Spain [20]. In summary, promoter regions of the scanned genes were interrogated for TF-specific motifs according to the TRANSFAC curated TFBS database [21]. DCGs with enrichment for a TFBS as well as the complete original modules were compared to the rest of the genome in order to detect significantly overrepresented Gene Ontology (GO) terms (levels from 4 to 7) using the FatiGO tool available in the Babelomics v4.3.

### 2.3. Selection of Candidates

Five TFs out of the significant ones in the enrichment analysis were selected for biological validation of our strategy based on the previous literature and the repetition of the terms in at least three different modules. For each TF, we selected up to three target genes from the DCGs sets with enrichment for that particular TF, prioritizing those with conserved binding sites and/or positive chromatin immunoprecipitation-sequencing (ChIP-seq) data at promoters and/or H3K27Ac-rich regions, according to the ENCODE data from the University of California Santa Cruz (UCSC) genome browser (https://genome.ucsc.edu/). Revision of the literature helped to further prioritize those genes that could be more relevant to CD pathogenesis, and *CISD2*, *HDAC4* and *WDR43* were chosen for IFN regulatory factor-1 (IRF1); *AKTIP*, *NAMPT* and *TPK1* for CAMP Responsive Element Binding Protein 1 (CREB1); *CRTAM*, *PLLP* and *RFX5* for ETS domain-containing protein (ELK1), and *WNT11* for NFKB1. Additionally, target genes that were not present in the DCGs sets but are well defined targets of the selected TFs were also included. Putative targets were sorted according to the number of TFs that are controlling them (based on the human/mouse/rat conserved binding sites from the UCSC genome browser tables utility), and the most specific target genes for the four candidate TFs were selected, namely *CXCL11* and *BATF2* for IRF1; *ISG15* and *HIST1H4C* for CREB1; NKG7 and *RAB17* for ELK1; and *GSTA4*, *TFF1* and *PLAUR* for NFKB1.

### 2.4. Patients and Biopsies

Biological confirmation of the regulatory elements proposed in silico was performed in duodenal biopsies from 28 newly diagnosed CD patients and 20 non-celiac controls diagnosed at the Cruces University Hospital Pediatric Gastroenterology Unit (Barakaldo, Spain). CD was diagnosed according to the European Society of Pediatric Gastroenterology, Hepatology and Nutrition (ESPGHAN) criteria in force at the time participants were recruited, and included anti-gliadin and/or anti-transglutaminase antibody determinations as well as a confirmatory small bowel biopsy. The study was approved by the Institutional Boards (Cruces University Hospital codes CEIC-E13/20 and CEIC-E16/46 and Basque Clinical Trials and Ethics Committee code PI2013072) and informed consent was obtained from patients or their parents.

Biopsy samples were immediately stored in liquid nitrogen until RNA was extracted using the NucleoSpin miRNA kit (Macherey-Nagel, Düren, Germany). Eluted RNA was quantified using a Nanodrop spectrophotometer (Life Technologies, Carlsbad, CA, USA, Thermo Fisher Scientific Inc., Waltham, MA, USA) and adjusted to 100 ng/μL in elution buffer.

### 2.5. Cell Culture and Stimulation

The C2BBe1 cell line, a subclone of the human intestinal Caco-2 that although derived from a colon adenocarcinoma, represents the best available in vitro model of absorptive enterocytes and it is widely used as an in vitro model in CD [22], was purchased from ATCC (ATCC, Manassas, VA, USA) and maintained in DMEM (Dulbecco’s modified Eagle’s medium) supplemented with 10% heat inactivated FBS (fetal bovine serum), 1% non-essential amino acids, and 1% penicillin-streptomycin in tissue culture flasks (Corning Costar, Cambridge, MA, USA). Cells were cultured at 37 °C in a humidified 5% CO_2_ atmosphere. Culture medium was changed every 2–3 days and cells were sub-cultured after reaching 70–80% confluence. For in vitro stimulation of cultured cells, pepsin-trypsin digests of gliadin (PT-G) (Sigma-Aldrich, St. Louis, MO, USA) and BSA (bovine serum albumin) (Thermo Fisher) (PT-BSA) were prepared as described previously [8]. Cells were exposed to 1 mg/mL PT-G or PT-BSA at 37 °C and 5% CO_2_ for 4 h.

### 2.6. Reverse Transcription and Quantitative PCR

To study the expression of candidate TFs and target genes, RNA was converted to complementary DNA (cDNA) using the SuperScript VILO cDNA Master Mix (Thermo Fisher), following the manufacturer’s protocol. Gene expression levels were measured by real-time qPCR on a Fluidigm BioMark dynamic array system (Fluidigm Corporation, San Francisco, CA, USA) using commercially available TaqMan Gene Expression assays (Supplementary Table S1). The expression of the housekeeping gene *TBP* was simultaneously quantified in each experiment and used as an endogenous control. All assays were performed in duplicate and expression levels were calculated using the 2^−ΔCt^ method as previously described [23] relative to the housekeeping gene *TBP*. Results were normalized to the average expression value of control samples.

### 2.7. Western Blot Analyses

Nuclear and cytoplasmic protein extracts of cells incubated with PT-G/PT-BSA for 4 h were isolated using a commercial Nuclear Extract Kit (Active Motif, Carlsbad, CA, USA) and protein concentrations were measured using the Pierce BCA Protein Assay Kit (Thermo Scientific, Waltham, MA, USA). Protein preparations were heat-denatured at 95 °C for 5 min, loaded into sodium dodecyl sulfate-polyacrylamide gel electrophoresis (SDS-PAGE) gels and transferred to nitrocellulose membranes using the Trans-Blot Turbo Transfer (BioRad, Hercules, CA, USA). After transfer, membranes were blocked in 5% non-fat milk in Tris-buffered saline with 0.05% Tween (TBST) and incubated overnight at 4 °C with the following primary antibodies: anti-HDAC1 (1:10,000 dilution; ABCAM, Cambridge, UK), anti-ELK1 (1:500), anti-NFκB p10550 (1:400), anti-CREB1 (1:1000) anti-IRF1 (1:1000) and anti-α-tubulin (1:5000 dilution, Sigma-Aldrich) followed by an incubation of 1 h with HRP (horseradish peroxidase) conjugated anti-rabbit Immunoglobulin G (IgG) (1:2000) and anti-mouse IgG (1:1000) secondary antibodies (Jackson ImmunoResearch Laboratories, Inc., West Grove, PA, USA). Proteins were visualized using SuperSignal West Femto Maximum Sensitivity Substrate (Thermo Fisher) on a ChemiDoc MP system.

### 2.8. Statistical Analyses

Default parameters in Babelomics v4.3 were used (*p* < 0.05, adjusted by FDR) to identify overrepresented TFBS and GO terms. Statistic calculations were performed in GraphPad Prism 5 (GraphPad Software, La Jolla, CA, USA). Gene expression levels in biopsies from the CD and control groups were compared using Mann Whitney test.

## 3. Results

### 3.1. Identification of Coexpressed Gene Modules and Differentially Coexpressed Genes in Celiac Disease

**Figure 1:**
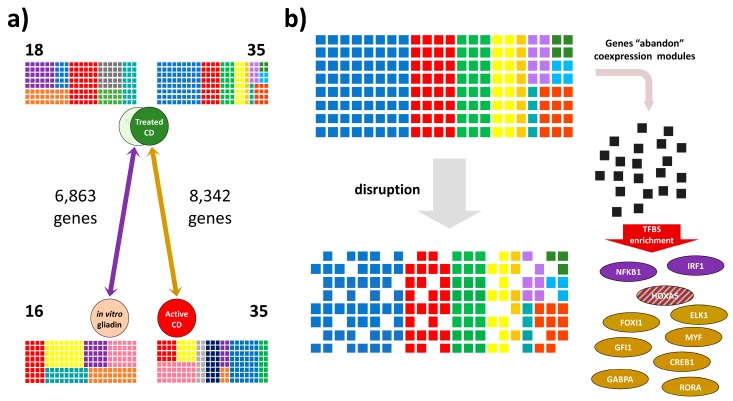
Co-expressed gene modules and differentially co-expressed genes in response to gliadin; (**a**) Representation of co-expression modules of acute (purple arrow) and chronic (golden arrow) response to gliadin. Each square represents a gene, while genes forming a co-expression module share a color. The number of genes used for the construction of modules is indicated for each experiment, and the numbers in bold represent the amount of modules identified in each experiment and condition; (**b**) Representation of differentially co-expressed genes (DCGs; black squares) and enrichment analysis for transcription factor binding sites (TFBS). Three transcription factors (TFs) were identified in the acute experiment (purple) and 8 TFs in the chronic experiment (gold).

To study how gluten alters co-expression in CD, we reanalyzed two expression microarray experiments previously performed in our laboratory; the effect of in vitro culture with (or without) 10 µg/mL gliadin of biopsy portions from CD patients on GFD, and the effects of chronic exposure to gliadin, comparing biopsies from CD patients at diagnosis and on GFD [14,15]. In the tissue-culture experiment 6863 genes were assigned to 18 modules in the GFD biopsies incubated without gliadin and to 16 modules after in vitro gliadin challenge. In the long-term exposure experiment 8342 genes were included in 35 modules in biopsies from GFD patients, and in another 35 modules in active CD (Figure 1A).

We searched for differentially co-expressed genes or DCGs, i.e., genes that change their relationship with their original co-expression module partners when comparing with vs. without gliadin situations in each of the two experiments. In the in vitro challenge experiment, co-expression was disrupted in 21.71% of the genes that had belonged to modules in the no-gliadin situation, and in 9.93% of those that were co-expressed in the stimulated group. In the long-term exposure experiment, the loss of co-expression affected more genes from the modules in the GFD situation than those originating from active disease co-expression modules (14.68% vs. 12.60%, respectively).

### 3.2. Transcription Factor Binding Site and Gene Ontology Enrichment Analyses in Differentially Co-Expressed Genes

DCGs of the 34 gene modules of the tissue-culture experiment and the 70 gene modules coming from the long-term experiment were further analyzed. In the tissue-culture experiment, DCGs from two modules showed TFBS enrichment: a module from biopsies incubated without gliadin was enriched for HOXA5 binding sites (Magenta module, Supplementary Table S2), and another module from the gliadin-challenged condition presented HOXA5, IRF1 and NFKB1 TFBS (Purple module, Supplementary Table S3). In the long-term experiment, one DCG-set in a module from the GFD-treated group showed TFBS enrichment for GFI1, FOXI1, ELK1, CREB1, GABPA, HOXA5, MYF and RORA (Brown module, Supplementary Table S4) (Figure 1B).

Several of the candidates found have been previously related to inflammation, like IRF1 [24] and CREB1 [25] or to other pathways associated with CD, including the NFκB pathway, as NFKB1 [9]; and Tight Junctions, like ELK1 [26]. Due to their implication in inflammation and CD, those TFs were selected to perform further analyses. On the other hand, as significant enrichment for HOXA5 targets was observed in different modules of both the acute and the chronic response experiments, HOXA5 was also selected.

**Figure 2:**
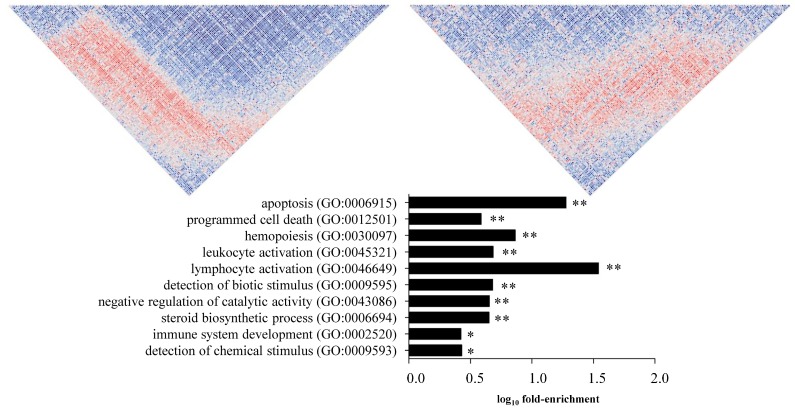
Co-expression and Gene Ontology (GO) annotation studies in DCGs. Co-expression pattern changes of DCGs with enrichment for CAMP Responsive Element Binding Protein 1 (CREB1), ETS domain-containing protein (ELK1) and homeobox protein Hox-A5 (HOXA5) binding sites, from treated celiac disease (CD) to active CD (chronic experiment). Each small square represents the *P*-value of the correlation between the expression levels of a specific gene pair (red-blue scale represents positive to negative Spearman correlation). Below, the top 10 GO annotations for those DCGs compared to the whole-genome. GO terms are indicated in the Y axis. The X axis shows the log_10_ fold-enrichment (ratio between the percentage of genes annotated with the GO term in the test set and the number of genes annotated with such term in the whole-genome (*** *p* < 0.001, ** *p* < 0.01 and * *p* < 0.05)).

To ascertain whether those genes escaping together the co-expression modules in which they were originally found and showed TFBS enrichment according to our analysis shared any kind of function or biological significance, we performed GO term enrichment analyses. We did not observe any particular enrichment in the genes escaping the Magenta module, while DCGs from the Purple and the Brown modules showed several terms related to the disease, including lysosomal transport [27] (GO:0007041), apoptosis (GO:0006915), lymphocyte activation (GO:0046649), immune system development (GO:0002520). Moreover, when these DCGs were removed from their original modules, no GO terms were identified (Figure 2) (Figure S2).

### 3.3. Altered Candidate Transcription Factors Expression in Celiac Disease

Our in silico analysis had proposed several TFs, namely HOXA5, IRF1, NFKB1, CREB1 and ELK1 that could underlie the disruption of co-expression among genes functionally implicated in CD, so we analyzed those TFs in an independent cohort of CD patients and controls.

**Figure 3:**
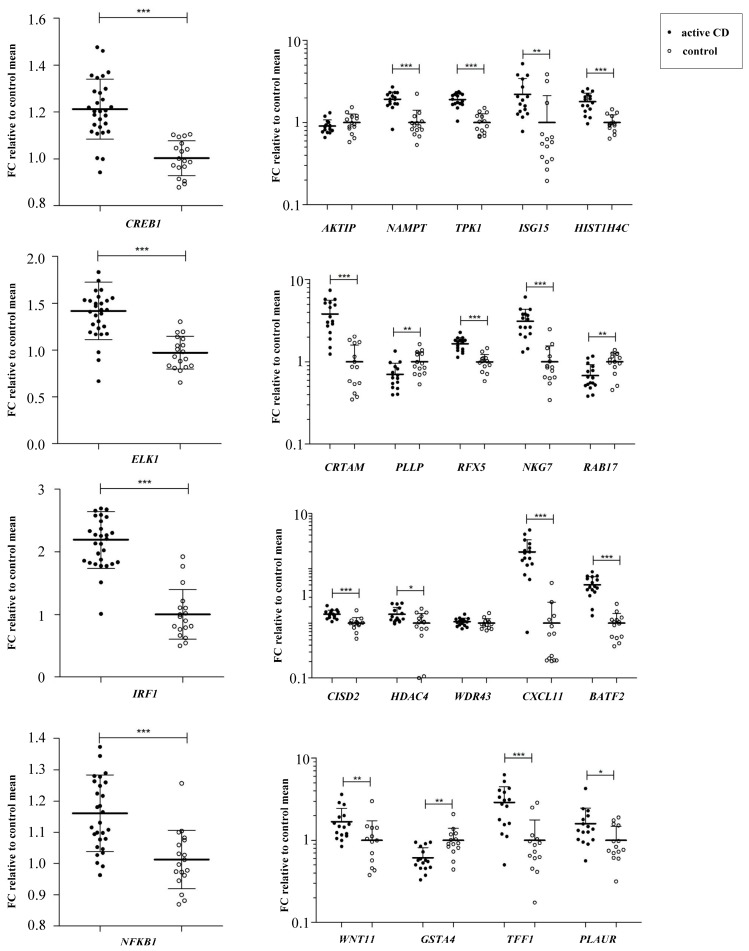
Gene expression analysis. Gene expression analysis of TFs identified by WGCNA (active CD *n* = 30, control *n* = 18) and their target genes (active CD *n* = 16, control *n* = 14) in duodenal biopsies. Data are expressed as mean ± SD (standard deviation) (*** *p* < 0.001, ** *p* < 0.01 and * *p* < 0.05).

Expression analyses showed that *IRF1*, *ELK1*, *NFKB1* and *CREB1* were significantly upregulated in disease. Additionally, out of 19 genes that were targets for those TFs, 17 were differentially expressed (*p* < 0.05) in biopsies from active CD patients (Figure 3).

### 3.4. Candidate Transcription Factors in C2BBe1 Cells

**Figure 4:**
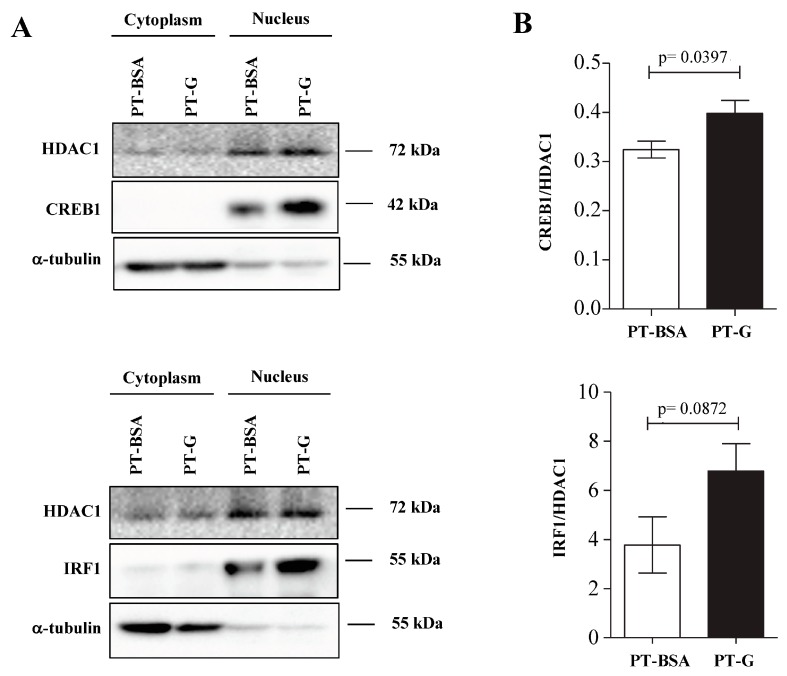
In vitro characterization of selected TFs. (**A**) The translocation of IFN regulatory factor-1 (IRF1) and CREB1 to the nucleus was analyzed by Western blot in 3 independent experiments upon stimulation with pepsin-trypsin digested gliadin (PT-G) or pepsin-trypsin digested BSA (PT-BSA) as control; (**B**) Band intensity was quantified and normalized to the intensity of the band corresponding to HDAC1. Mean values were compared with a *t*-test. Data are expressed as mean ± SEM.

We studied the translocation of the differentially-expressed TFs into the nucleus in the C2BBe1 cell line. Particularly, we investigated the cellular localization of IRF1, NFKB1, ELK1 and CREB1 using western blot analysis of the nuclear and cytoplasmic fractions of C2BBe1 cells exposed to PT-G or PT-BSA (control) during 4 h. PT-G induced a consistent increase of CREB1 and IRF1 in the nucleus, suggesting the activation of these TFs upon gliadin challenge (Figure 4).

## 4. Discussion

Co-expression analyses identify groups of co-transcribed genes that might share common regulatory elements, which in turn could be responsible for the changes in expression and co-expression that occur upon different stimuli. It has been shown that gliadin is able to disrupt co-expression in several groups of genes and provoke the coordinated response in others [7,9]. However, co-expression analyses performed in CD so far have been limited to a small number of genes or pathways, and to a single work that studied genome-wide co-expression in peripheral blood [13]. This is the first whole genome co-expression analysis that tests the effects of gliadin in duodenum biopsies of CD patients, and we presently identify regulatory elements that could play a role in the development of the disease.

First of all, co-expression was analyzed in expression microarray experiments resembling the acute and chronic effects of gliadin in the celiac gut [14,15]. We observed a complete reorganization of co-expressed gene groups in both experiments, but the acute insult provoked more drastic co-expression changes. As gene regulation plays a key role in complex disease pathogenesis, identification of the potential regulatory elements involved in this interruption of coordination could be a good strategy to understand better CD pathogenesis and develop possible future medical applications.

Once differentially co-expressed genes had been identified, enrichment analysis for TFBS and GO terms was performed in order to find potential co-expression modifiers. TFs are key molecules that control gene expression, and approximately 10% are implicated in diverse human diseases [28]. TFBS enrichment analyses revealed for the importance of several TFs in three DCGs-sets, two in the acute experiment (coming from the Magenta and Purple modules) and one in the chronic experiment (coming from the Brown module), suggesting that there might be additional mechanisms other than TFs that control co-expression disruptions in CD. GO enrichment was studied in the DCG-sets that showed TF motif enrichments with the final aim of ascertaining whether those genes showed any common biological function. In particular for the DCGs in the Brown module, the most significant GO annotations were related to immune system and apoptosis processes, both of them being key elements in CD pathogenesis since CD is an autoimmune disease in which apoptotic activity is increased in the intestinal mucosa [1,29]. When GO annotations where studied in the DCGs coming from the Purple module, several CD-related GO terms such as vasodilation [30] or lysosomal transport [27] were found. This could suggest that gliadin induced co-expression changes could affect the proper functioning of the mentioned biological processes. Finally, when the original Brown, Purple and Magenta gene modules were studied as a background, many GO annotations were identified. Interestingly, some of them were related to CD, namely cell-cell adhesion involved in intestinal permeability [31], inflammation mediators (protein kinase cascade) [32], or apoptosis [29], but when the DCGs were removed from their original modules, no significant GO annotations remained. These results confirm a pivotal role of the DCGs in the functions defined for the modules where they were first identified and support the biological relevance of our enrichment analyses.

Five TFs were prioritized for biological validation of our in silico approach. IRF1 and CREB1, both of them related to the innate and adaptive immune responses [24,25] showed increased mRNA expression in active patients compared to the control group. This is the first work to show overexpression of CREB1 in CD, while the increase of IRF1 confirms previous reports [33]. On the other hand, overexpression of the NFKB1 subunit in disease is in accordance to the involvement of the NFκB pathway in CD [9]. ELK1 was overexpressed at mRNA level in active CD, a TF that is known to be involved in intestinal permeability [26], an important feature of CD [31]. More interestingly, many of their target genes followed a trend in disease concordant with the activatory patterns observed for the TFs themselves.

As DCGs were identified because of gliadin-induced co-expression changes, we wanted to know whether the immunogenic compound could alter those TFs at the protein level. Upon gliadin stimulation, increased nuclear translocation of CREB1 and IRF1 was observed in the C2BBe1 cell line. In a recent work, IRF1 was upregulated by CD-associated bacteria [34], so that together with our results, we could suggest an increase of IRF1 activity in situations that promote the disease. There are no previous data on CREB1 in CD, but it has been related to other diseases like human colorectal cancer, where CREB1 acts as a TF for tumor driver *RRM2*, or glioblastoma, where CREB1 acts as a mediator of the induction of TGFβ2 [35,36]. This increase in nuclear translocation suggests a role for gliadin in the upregulation of their target genes through TF activation, also in non-celiac individuals.

Even though mechanistic studies will be necessary to completely understand the molecular and cellular mechanisms underlying our observations, our work shows that co-expression studies can complement classical differential expression analyses, and are particularly useful for the identification of regulatory elements that could be relevant to human diseases. Moreover, these approaches can be applied to freely available genome-wide datasets by reusing raw experimental results to uncover novel layers of genomic information.

## Supplementary Materials

The following are available online at http://www.mdpi.com/2073-4425/9/5/245/s1. Figure S1: Flowchart of the methodology used for the microarray and bioinformatics analyses; Figure S2: (A) Co-expression pattern changes of DCGs with enrichment for HOXA5 binding sites, from basal CD to gliadin-challenged CD; (B) Co-expression pattern changes of DCGs with enrichment for IRF1, HOXA5 and NFKB1 binding sites, from gliadin-challenged CD to basal CD. Each small square represents the *p* value for the correlation of the expression level in a specific gene pair. Red-blue scale represents positive to negative Spearman correlation. Below, the top 10 GO term annotations for those DCGs with respect to the genome; Table S1: TaqMan assays for expression analysis; Table S2: Gene expression data for the genes co-expressed in the Magenta module (tissue-culture experiment). B = biopsies in basal conditions; G = gliadin-stimulated biopsies. Table S3: Gene expression data for the genes co-expressed in the Purple module (tissue-culture experiment). B = biopsies in basal conditions; G = gliadin-stimulated biopsies. Table S4: Gene expression data for the genes co-expressed in the Brown module (long-term experiment). T = treated, inactive CD patients; D = CD patients at diagnosis.
