# Supplementary material for: Transcription Factor Binding Site Enrichment Analysis in Co-Expression Modules in Celiac Disease
Source: Genes (Basel). 2018 May 10;9(5):245. doi: 10.3390/genes9050245 (PMC5977185; doi:10.3390/genes9050245)
Supplement: Supplementary file 1 [file genes-09-00245-s001.zip › genes-300110- Supplementary resubmitted/Figure S1.pdf]

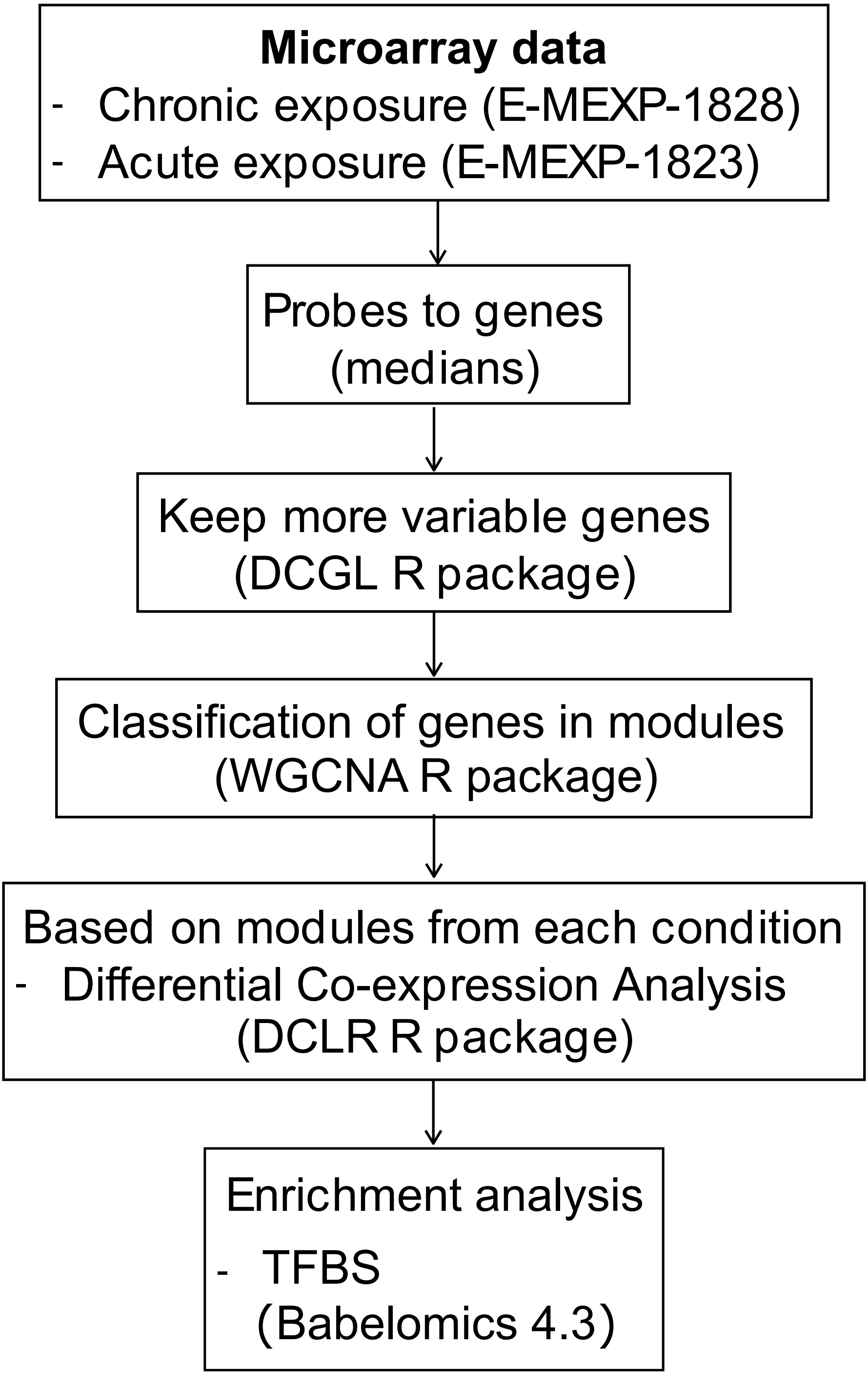
Microarray data
- Chronic exposure (E-MEXP-1828)
- Acute exposure (E-MEXP-1823)
Probes to genes (medians)
Keep more variable genes (DCGL R package)
Classification of genes in modules (WGCNA R package)
Based on modules from each condition
- Differential Co-expression Analysis (DCLR R package)
Enrichment analysis
- TFBS (Babelomics 4.3)
