# Supplementary figures and images for: Transcription Factor Binding Site Enrichment Analysis in Co-Expression Modules in Celiac Disease
Source: Genes (Basel). 2018 May 10;9(5):245. doi: 10.3390/genes9050245 (PMC5977185; doi:10.3390/genes9050245)

A

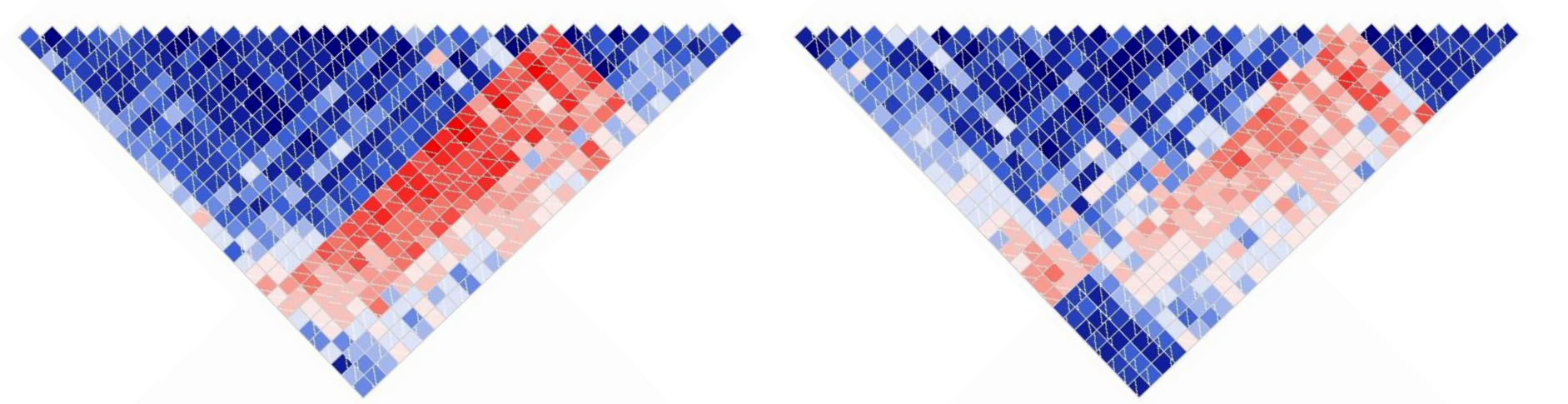

B

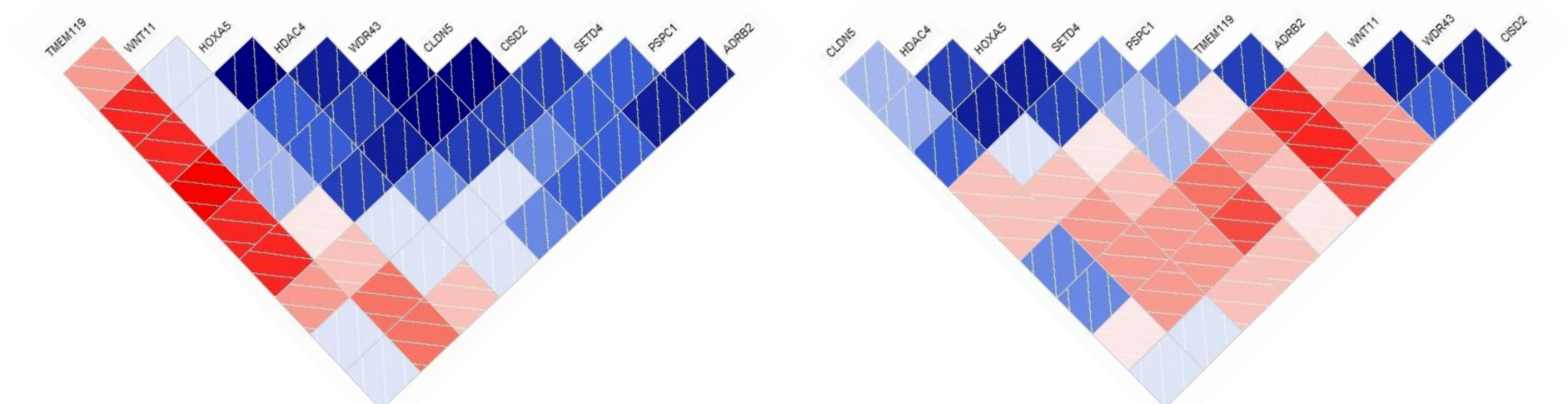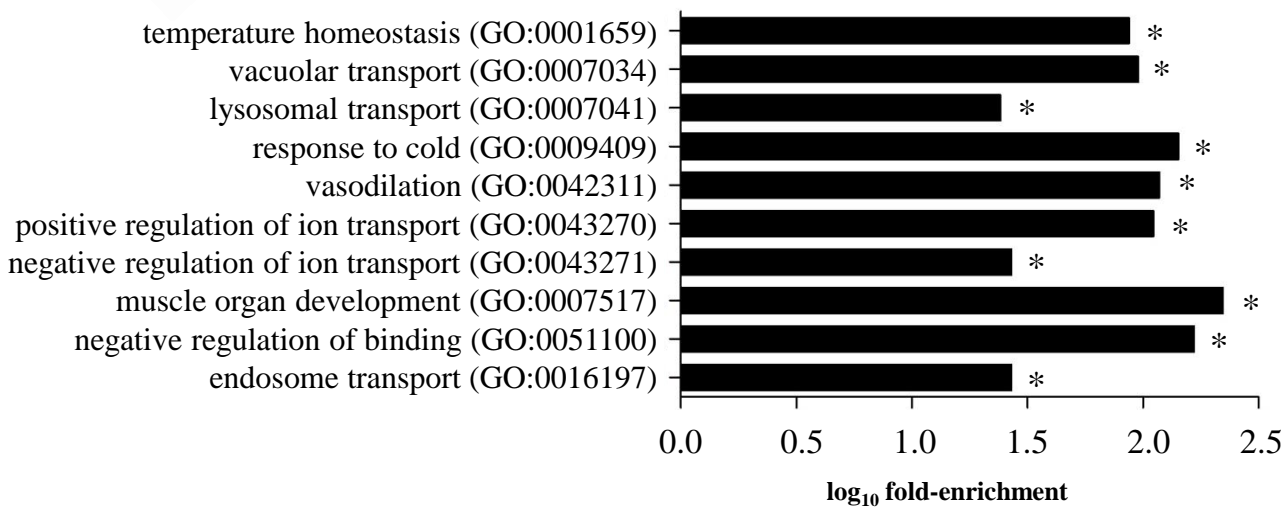

Supplement: Supplementary file 1 [file genes-09-00245-s001.zip › genes-300110- Supplementary resubmitted/Figure S2.pdf]
